# Spondyloarthritis: Matrix Metalloproteinasesas Biomarkers of Pathogenesis and Response to Tumor Necrosis Factor (TNF) Inhibitors

**DOI:** 10.3390/ijms18040830

**Published:** 2017-04-14

**Authors:** Stefania Moz, Ada Aita, Daniela Basso, Roberta Ramonda, Mario Plebani, Leonardo Punzi

**Affiliations:** 1 Laboratory Medicine, Department of Medicine—DIMED, University of Padova, Via Giustiniani 2, 35128 Padova, Italy; stefania.moz@studenti.unipd.it (S.M.); ada.aita@tiscali.it (A.A.); mario.plebani@unipd.it (M.P.); 2 Rheumatology Unit, Department of Medicine—DIMED, University of Padova, Via Giustiniani 2, 35128 Padova, Italy; roberta.ramonda@unipd.it (R.R.); leonardo.punzi@unipd.it (L.P.)

**Keywords:** spondyloarthritis (SpA), inflammation, tumor necrosis factor (TNF), TNF inhibitors, matrix metalloproteinases (MMPs), biomarkers

## Abstract

The term spondyloarthritis (SpA) is used to describe a group of multifactorial chronic inflammatory diseases characterized by a predisposing genetic background and clinical manifestations typically involving the sacroiliac joint. The absence of pathognomonic clinical and/or laboratory findings generally results in a delay in diagnosis and, consequently, in treatment. In addition, 20–40% of SpA patients are non-responders to tumor necrosis factor (TNF) inhibitor therapies. Given these considerations, it is important to identify biomarkers that can facilitate the diagnosis and assessment of disease activity. As inflammation plays a key role in the pathogenesis of SpA, inflammatory mediators have been investigated as potential biomarkers for diagnosing the disease and predicting response to therapy. Some investigators have focused their attention on the role of matrix metalloproteinases (MMPs), which are known to be markers of synovial inflammation that is generated in the joint in reaction to inflammatory stimuli. Several studies have been carried out to verify if serum MMPs levels could be useful to diagnose SpA, to assess disease severity, and to predict response to TNF inhibitor therapy. The current review focuses on MMPs’ role in SpA pathogenesis, diagnosis and therapeutic implications.

## 1. Introduction

Affecting the sacroiliac joint, spondyloarthritis (SpA) is a family of chronic inflammatory diseases that generally present at a young age (<45 years) and that are characterized by a heavy symptomatic burden and loss of function during patients’ productive years. Their prevalence is low in South-East Asia (0.20%; 95% Confidence Interval (CI): 0.00–0.66), high in Northern Arctic communities (1.61%, 95% CI: 1.27–2.00) and in North America (1.35%; 95% CI: 0.44–2.73), and intermediate in European populations (0.54%; 95% CI: 0.36–0.78) [1]. In 2009 and 2011, the Assessment of Spondyloarthritis International Society (ASAS) developed the criteria for defining axial (axSpA) and peripheral (pSpA) spondyloarthritis, depending on the sites predominantly manifesting the disease. Peripheral manifestations may present before, at the same time, or after the diagnosis of axSpA.

Ankylosing spondylitis (AS) is the prototype axSpA, and psoriatic arthritis (PsA) is a form of arthritis affecting individuals with psoriasis. Depending on the presence or absence of structural damage of the bone detectable on X-ray scans, axSpA is further subdivided into two main groups: radiographic and non-radiographic axSpA (nr-axSpA). Peripheral SpA is typically a mono- or oligo-articular (less than five joints) arthritis predominantly involving the lower limbs and often characterized by enthesitis and dactylitis.

The presentation of SpA is further complicated due to the association of extra-articular manifestations, such as uveitis, psoriasis and inflammatory bowel diseases (IBD). Averaging a delay ranging from 8 to 11 years, the diagnostic process is often laborious because of the absence of pathognomonic clinical and/or laboratory findings [2], thus causing late onset of treatment. According to the ASAS criteria for SpA diagnosis, the disease can be suspected in the event of chronic back pain lasting at least three months in a patient younger than 45 years of age at onset. The diagnosis is confirmed when there is imaging evidence of sacroilitis and at least one spondyloarthritis feature (Table 1) or, in the absence of the former, of at least two spondyloarthritis features in HLA-B27 positive patients, the genetic haplotype frequently associated with AS and, less frequently, with PsA [3,4].

An important factor contributing to the delay normally characterizing SpA diagnosis is linked to the absence of specific blood biomarkers. Commonly used inflammatory markers such as C-reactive protein (CRP) or the erythrocyte sedimentation rate (ESR) often fall within reference ranges in patients with inflammatory spine symptoms and nr-axSpA; high levels are associated with more severe AS (40–50% of patients) and are found in patients with acute exacerbations.

Sensitive and/or specific imaging or biological markers could aid clinicians to formulate an early diagnosis of the disease [3]. Biomarkers could also be used to classify disease activity, which is presently based almost exclusively on clinical indexes such as the Bath Ankylosing Spondylitis Disease Activity Index (BASDAI), which quantifies a patient’s self-assessment of symptoms such as fatigue, pain, swelling, axial and peripheral symptoms, enthesopathy, and duration and intensity of morning stiffness, the Ankylosing Spondylitis Disease Activity Score (ASDAS), the Maastricht Ankylosing Spondylitis Enthesitis Score (MASES), the Disease Activity Score-28 (DAS-28), the Bath Ankylosing Spondylitis Functional Index (BASFI), the Health Assessment Questionnaire (HAQ) and the Bath Ankylosing Spondylitis Metrology Index (BASMI) [5].

## 2. The Pathogenesis of Spondyloarthritis: Genetics, Inflammation and Immunity

Despite the numerous studies that are available in the literature, the pathogenesis of SpA is still not completely understood. SpA is a group of multifactorial diseases that result from a complex interplay between an inherited genetic background (mainly represented by the HLA-B27 haplotype) and environmental factors (infections, mechanical stress, abnormal intestinal microbiota) that lead to immune response dysregulation and autoinflammation [6]. As far as the most studied genetic markers are concerned, alleles at the HLA locus on chromosome 6p have proven to be the most informative. Associations between HLA and PsA have been demonstrated, particularly for class I alleles, at the B and C loci.

With respect to most rheumatic diseases, heredity plays a particularly important role in PsA. Approximately 15% of relatives of patients with psoriasis manifest PsA, and a further 30–45% have psoriasis. The allele most frequently associated to psoriasis is HLA-CW6, associated with more severe and early onset of both psoriasis and PsA. [7].

The HLA-B27 haplotype is closely associated with axSpA susceptibility, especially with AS. In fact, 80–90% of AS patients and 50–75% of patients with other SpAs are carriers of this haplotype, although the mechanisms underlying this striking association are only partially understood [8].The HLA-B27 gene consists of at least 132 different alleles coding for 105 protein subtypes (named HLA-B*27:01 to HLA-B*27:105). The most common subtypes associated with AS are HLA-B*27:02 in Mediterranean populations, HLA-B*27:04 in the Chinese population, HLA-B*27:05 in Caucasians, and B*27:07 in South Asian and Middle Eastern populations. The differences between HLA-B27 proteins encoded by AS-associated (B*27:05) and AS-non-associated alleles (B*27:09) regard: structural conformation, protein binding, thermodynamic stability and cell surface expression [9].

HLA-B27, which is involved in antigen presentation, plays a potential role in SpA pathogenesis because of its link with the immune response through mechanisms that have been explained by three main theories: the “arthritogenic peptide”, the “misfolding”, and the “cell surface HLA-B27 homodimers” hypotheses. The arthritogenic-peptide theory, which was formulated in 1990 [10], is the most accepted pathophysiological framework for SpA. It is based on the premise of a molecular mimicry between pathogenic antigens (e.g., intracellular bacteria or an ubiquitous virus) and cartilage/bone-derived self-peptides that activate cytotoxic T lymphocytes (CTLs) after HLA-B27 antigen presentation [9]. This hypothesis has been supported by the finding of autoreactive HLA-B27—restricted CTLs that recognize peptides derived from intracellular bacteria as well as uninfected healthy cells in the synovial fluid of AS patients [11], and by the observation that gastrointestinal or urogenital infections can trigger SpA. The theory has not, however, been entirely corroborated by the identification of an autoimmune arthritogenic peptide [12] or in animal models [13,14].

The other two hypotheses argue in favour of the theory that HLA-B27 plays an autoinflammatory role in triggering the innate immune responses [15]. The misfolded HLA-B27 heavy chains tend to accumulate in the endoplasmic reticulum (ER), triggering ER stress, which leads to the activation of the unfolded protein response (UPR) and the NF-κΒ pathway which, in turn, leads to the release of pro-inflammatory cytokines, such as TNF-α, IL-1, IL-6, mainly by monocytes/macrophages, thus favouring the inflammatory process [16]. Animal disease models argue both in favour and against this hypothesis [17,18]. The cell surface HLA-B27 homodimers hypothesis is, instead, based on the observation that HLA-B27 homodimers produced at the cell surface bind to specific receptors expressed on NK cells, T-lymphocytes, and myelomonotic cells producing an immunomodulatory effect [19]. This hypothesis has been supported by the finding that the number of NK and CD4^+^ T-cells expressing a receptor that recognizes HLA-B27 homodimers but not heterodimers is higher in HLA-B27 positive patients [20]. Although HLA-B27 is the most important genetic factor predisposing to AS, it contributes to only 20–30% of the total heritability, and less than 5% of HLA-B27 carriers in the general population develop the disease. The major histocompatibility complex (MHC) accounts for approximately 40–50% of the genetic risk, and MHC genes other than HLA-B27, such as HLA-B (B*40:01), appear to be associated with AS [21]. To date, over 41 non-MHC genes have been identified as associated with AS. Although each gene variant determines a very small percentage of risk [21], many appear to be involved in the innate and adaptive immune response, supporting the hypothesized de-regulated immunity role in SpA pathogenesis. In support of this hypothesis, the synovial membrane of patients with SpA appears to be infiltrated by cells of the innate (neutrophils and macrophages) and adaptive (dendritic cells, lymphocytes, microanatomical features of germinal center formations capable of antibody production, oligoclonal expansion of CD8^+^ T cells in the synovial fluid) immune response.

One of the most relevant immune cell subset involved in SpA pathogenesis appears nevertheless to be represented by Th17 T cells, and this would explain the promising results that have been obtained with drugs targeting the IL17/IL12/IL23 pathway [4,22,23]. Over the past decade, SpA treatment has taken great strides thanks to tumor necrosis factor (TNF) inhibitors. TNF-α, which is a pro-inflammatory cytokine that is primarily involved in homeostatic functions and inflammation, is produced by various cell types including mononuclear and polymorphonuclear cells that favour the inflammatory process by activating critical intracellular signalling pathways (NF-κB, c-Jun and apoptosis) and cytokine release [24,25]. To date, five biologic agents targeting TNF-α have been approved for the treatment of rheumatoid arthritis, psoriatic arthritis, ankylosing spondylitis, juvenile idiopathic arthritis, IBD, psoriasis, and, most recently, hidradenitissuppurativa [25]. These drugs comprise four monoclonal anti-TNF agents (Infliximab, Adalimumab, Golimumab, Certolizumab) and the soluble tumor necrosis factor (Etanercept). Overall, these anti-TNF agents act by inhibiting TNF-α from binding to its receptors thus interfering with TNF-α signaling transduction pathways.

TNF inhibitors are highly effective in targeting different disease features, not only with regard to axial disease and peripheral arthritis but also enthesitis and extra-articular characteristics such as psoriasis or uveitis [26]. Treatment with anti-TNF agents leads to a significant improvement in work productivity (employment, sick leave) and physical activity (participation rate, hours/week, and physical intensity) [27]. It has also been demonstrated that the effectiveness of TNF inhibitors is maintained over several years’ time, and a variety of these agents seem to produce the same effect on different clinical manifestations [28].

Despite the findings confirming the therapeutic effectiveness of TNF inhibitors that has been noted, 20–40% of patients fail to respond. Some studies have demonstrated that in the event that the first anti-TNF agent utilized is ineffective, others can be tried as patients may respond to one while not to another. No valid biomarkers are as yet available to predict treatment failure with TNF inhibitors, an important consideration given the burden of these drugs for the health care systems. A cross sectional study analyzing the U.S. health care utilization in patients with IBD between 2010 and 2012 using insurance claim data demonstrated that biologics were the major cost component in IBD care (35% out of $3.9 billion), although their use was restricted to only 11% of patients [29].

## 3. Pathogenesis of Spondyloarthritis: The Extra-Cellular Matrix and Matrix Metalloproteinases

Innate and adaptive immune cells typically infiltrate inflamed joints and are in direct contact with the extra-cellular matrix (ECM), which is composed of a complex mixture of insoluble molecules including collagens, laminins, fibronectin, entactin/nidogen and heparan sulphate proteoglycans. The ECM provides a solid support for the cells and acts as a reservoir for cytokines and growth factors; it also harbours cryptic information within molecules that compose the ECM network [30].

The regulated turnover of the molecules that compose the ECM is crucial for the interaction of individual cells with the surrounding environment, for proper physiological function, and for the development of the multicellular organisms [31].

The ECM present in the synovial membrane and cartilage is a key component of the joints. Far from being an inert structure, it undergoes continuous re-modeling which, in a homeostatic equilibrium, guarantees joints’ integrity. In the cartilage, the ECM is mainly composed of collagens (type II, but also types IX and XI collagen) and proteoglycan aggrecan. Chondrocytes, with a cell volume approximating 2% of the total cartilage volume, are mainly involved in its turnover and re-modelling. Further elements of the cartilage matrix are leucine-rich proteoglycans, including decorin, fibromodulin and biglycan [32].

**Figure 1:**
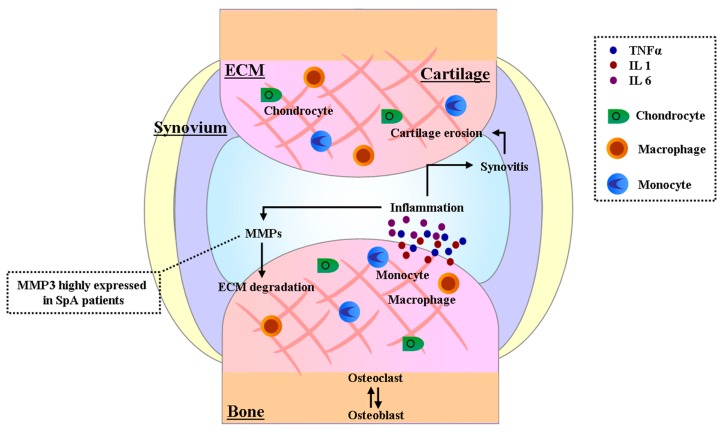
Cartilage re-modeling in the Spondyloarthritis SpA pathogenesis. The modulation of cartilage re-modeling in SpA patients is due to inflammatory conditions where monocytes and macrophages release inflammatory molecules (tumor necrosis factor alpha (TNFα), interleukin 1 (IL 1) and interleukin 6 (IL 6)). The inflammatory mediators also induce the production of metalloproteinases (MMPs), the main enzymes involved in extra-cellular matrix degradation (ECM).

The cartilage re-modeling process is conducted entirely by a single cell type, namely the chondrocyte, while osteoclasts play a prominent role in the bone re-modeling cycle. Cartilage model systems designed to study rheumatoid arthritis or osteoarthritis have shown that in response to inflammatory stimuli, such as IL-1β, chondrocytes rapidly induce the release of proteoglycans, which are quickly re-synthesized [33,34]. In a process that represents an irreversible step in cartilage destruction, collagen degrades at a faster rate with respect to proteoglycans. Similar mechanisms may underlie the evolution of sacroiliac joint destruction in spondyloarthritis. Collagen and bone degradation is mainly caused by matrix metalloproteinases (MMPs), a family of zinc-dependent endopeptidases that cleave most, if not all, ECM components (Figure 1).

Metalloproteinases belong to a superfamily of zinc-dependent proteases known as metzincins. The superfamily of zinc-dependent proteases includes also: adamlysins (ADAMs), ADAMs with thrombospondin-like motifs (ADAMTSs), astacins, serralysins and pappalysins [35]. The term “matrix metalloproteinase” defines these enzymes emphasizing the dependence of their activity on metal ions and their ability to degrade the structural proteins of the extracellular matrix [31]. In addition to the substrates of the extracellular matrix, the metal proteinases also break down some cell surface molecules and other peri-cellular proteins.

The MMPs family is composed of 28 members which are classified into collagenases, gelatinases, membrane-type metalloelastases (MT-MMPs), stromelysins, matrilysins, anamelysins, and unclassified subgroups [36]. The classification is based on the substrate specificity and the cellular localization of each MMP, which consists of several functional domains including: the N-terminal signal peptide domain responsible for secretion; the propeptide domain containing a conserved cysteine residue which interacts with the zinc ion in the catalytic site, and the haemopexin-like domain (except MMP7, MMP23 and MMP26) of a propeller blade structure.

MMPs are maintained in their inactive form through the coordination of the catalytic zinc with the conserved cysteine present within the propeptide domain (the highly conserved sequence [...PRCGXPD...]). Upon removal of the propeptide by proteases or disruption of the cysteine-zinc bond, the activation of the enzymes occurs through the cysteine-switch mechanism [37,38]. MMPs’ protein levels and enzyme activity are regulated by several mechanisms including: gene expression, zymogen activation, compartmentalization and inhibition of active enzymes [39]. Gene expression of most MMPs is regulated at the transcriptional level by various growth factors, hormones, cytokines, chemokines, tumor promoters and cell–cell or cell–ECM interactions [40]. At the post transcriptional level, MMPs expression is regulated through mRNA stabilization [41], and the contribution of epigenetic modification has also been recently uncovered [42].

The cleavage of the propeptide domain occurs through different mechanisms, i.e., proteolysis (Ser proteases or other MMPs), oxidation of the cysteine residue (reactive oxygen species including those released by polymorphonuclear neutrophils), structural perturbation by denaturants, or low pH [43,44,45,46]. The activity of MMPs is controlled by adhesion to structural ECM components or to cell receptors, such as integrins and lipids; MMPs cellular endocytosis followed by lysosomal degradation or recycling also contributes to regulating their activity [43,44,45,46]. The α2-macroglobulin and tissue inhibitors of metalloproteinases (TIMPs), the two major inhibitors of MMPs, significantly contribute to regulating MMPs activity in biological fluids such as blood and in extracellular space [31,45,47].

MMPs are produced by various types of cells, which include inflammatory, stromal, epithelial and endothelial cells. We have recently demonstrated that metalloproteinases, MMP8 and MMP9 in particular, are produced by peripheral blood mononuclear cells if they are stimulated by calprotectin, the S100A8/S100A9 heterodimer [48]. This finding seems potentially relevant to the pathophysiology of arthritis since peripheral blood mononuclear cells represent a dynamic cellular population that can infiltrate the inflamed tissues where they differentiate into inflammatory macrophages and thereby contribute to sustaining the inflammatory process. Calprotectin has been found to be increased in the sera of patients with axSpA, in particular in those with worsening radiographic severity [21], while S100A8/S100A9 has been shown to play a critical role in synovial inflammation, bone erosion, and cartilage damage [49].

## 4. Matrix Metalloproteinases as Biomarkers in Spondyloarthritis

It has been reported that MMPs, and MMP3 in particular, are produced in response to cytokines in the joints, being more highly expressed in the synovial tissues of SpA patients than in peripheral blood mononuclear cells [50]. In view of these findings, studies have been performed in order to verify if serum MMPs level assessment can be used as a biomarker for diagnosing SpA, assessing disease severity, and predicting response to therapy with TNF inhibitors. The majority of studies focusing on MMP3 are in agreement with the finding of higher serum levels in SpA, in both AS and PsA subtypes, with respect to those in healthy controls, and these results are confirmed by a recent meta-analysis which demonstrated that increased serum MMP3 levels are associated to higher AS risk [51]. However, the serum MMP3 levels related to the development and progression of AS were found to be conditioned by different geographical and genetic factors. In fact, at the ethnicity-stratified analysis, the MMP3 levels were higher in Asians and Caucasians than in African AS patients [51]. Although higher MMP3 baseline values appear to be correlated with the severity of SpA assessed biochemically (CRP) or using the BASDAI or BASFI severity indices, and seem to suggest peripheral joint involvement, the correlation between MMP3 serum levels and disease activity indexes is sometimes contrastive [52,53,54,55,56,57,58,59,60,61,62,63,64]. MMP3 together with CTX-II (C-terminal cross-linking telopeptide of type II collagen) seem to be particularly useful to predict radiographic progression of AS patients treated conventionally, outperforming over baseline CRP levels and BASDAI [65].

## 5. Usefulness of MMPs in Monitoring Response to Tumor Necrosis Factor-Targeted Therapy

We are living in the era of targeted therapies, which allows us to obtain significant clinical response in patients with osteoarticular diseases, including SpA [4]. The main limitation of this medical improvement depends on the 20–40% rate of non-responders to this expensive treatment. The identification of biomarkers for good response to therapy is therefore a real need to reduce inappropriate treatments and also to save health care costs.

Baseline, but mainly time-dependent variations in MMP3 levels seem able to predict the response to TNF inhibitor therapy both in PsA and AS. In agreement with this, by studying 43 PsA patients under treatment with etanercept, we have previously found that only MMP3, not CRP or Vascular Endothelial Growth Factor serum levels, were reduced after six months of therapy despite higher baseline levels of all indices in patients with respect to the controls [55]. MMP3, therefore, has been suggested as a useful marker to evaluate the early response to therapy.

The role of MMP3 as a possible biomarker of response to anti-TNF therapy in PsA patients was also studied by Chandran et al. [66], who investigated 24 PsA patients with active disease treated with adalimumab. After 12 weeks of therapy, the authors found a marked reduction in serum MMP3 levels, but not in other potential biomarkers (C-propeptide of type II collagen, procollagen type I N-terminal propeptide, melanoma inhibitory activity, type II collagen neoepitopes Col2-3/4C_long mono_, cartilage oligomeric matrix protein, osteocalcin, N-terminal telopeptide of type 1 collagen, and pyridinoline cross-linked carboxy-terminal telopeptide of type I collagen) [66]. Other MMPs have been also proposed as biomarkers of response to therapy, such as MMP13, which expression in synovial tissue of PsA patients was found to be reduced after adalimumab treatment [67].

MMP3 was also suggested to be a useful biomarker for monitoring the success of anti-TNF therapy in AS patients. Decreased serum MMP3 after 36–52 weeks of treatment with adalimumab, infliximab or with etanercept predicted a good response to therapy and was correlated with CRP [60]. The successful treatment of inflammation might induce the repair of the structural damage characterizing AS patients, through new bone formation and the increase of bone mineral density. To support this notion, it was recently suggested that the decrease of systemic inflammation is associated with new bone formation in SpA patients during anti-TNF therapy [58].

Moreover, MMP3 has been associated with peripheral arthritis. Arends et al. [61], demonstrated that serum MMP3 levels after 3 and 12 months of etanercept treatment significantly decreased in male patients with concomitant peripheral arthritis, but not in male patients with only axial disease. MMP3 is in fact primarily produced by fibroblasts and macrophages in the peripheral synovial joint [61]. The authors also showed that serum MMP3 levels were higher in male than in female AS patients, according to Natoli et al., who demonstrated higher gene and protein expression of MMP3 induced by male sex testosterone [68]. Table 2 summarizes the results of studies focused on MMPs in diagnosis and therapy monitoring in SpA.

## 6. Therapeutic Implications of Targeting Matrix Metalloproteinases

The effect of MMP inhibitors for the treatment of SpA can be considered the starting point for future research projects. MMP activity could be inhibited by using the naturally occurring molecules involved in regulating its activity, namely the tissue inhibitors of metalloproteinases (TIMPs), a family of four members (TIMP-1, -2, -3, -4) which stechiometrically bind MMPs by a 1:1 ratio causing a reversible inhibition of activity. While all TIMPs have tissue-specific expression and are present in a soluble form, only TIMP-3 is sequestered in the ECM. Moreover, unlike the other TIMPs, TIMP-3 is characterized by a broad inhibition spectrum including ADAMTS4 and ADAMTS5. Some investigators have suggested that the engineered TIMP-3 molecule can be used as a therapeutic drug for arthritis [34]. A number of small new molecules targeting MMPs have been engineered and studied for their potential application in arthritis treatment. These include plant derivatives such as dehydrocorydaline, shown to inhibit both mRNA and protein levels of MMP7 and MMP9 [73], and caffeic acid, which inhibits MMP9 activity [74,75]. Moreover, using a computational/directed-evolution approach to protein engineering, Arkadash et al. engineered an N-TIMP2 mutant that is able to selectively inhibit MMP14, overcoming the main limitation of MMP inhibitors, i.e., numerous, serious side effects due to their broad, non-selective inhibitory effect on MMPs [76]. In an animal model of experimental arthritis, Kaneko et al. demonstrated that treatment with antibodies against MT1-MMP reduces cartilage degradation and disease progression and, importantly, the treatment was shown to act synergistically with anti-TNF treatment [77].

In conclusion, the assessment of MMPs in body fluids promises to be an accurate biomarker for the assessment of SpA, and drugs targeting MMPs potentially represent useful treatment strategies. Before either can be utilized in the clinical setting, however, procedures to assay these biomarkers must be standardized, and safe bioavailable drugs with no relevant side effects need to be developed and tested.

## Figures and Tables

**Table 1:** Clinical, biochemical and genetic features of spondyloarthritis.

Features of Spondyloarthritis According to the ASAS Criteria
Inflammatory back pain
Arthritis
Enthesitis
Uveitis
Dactylitis
Psoriasis
Chrohn’s disease/Ulcerative colitis
Good response to NSAIDs
Family history of spondyloarthritis
HLA-B27
Elevated CRP

ASAS = Assessment of Spondyloarthritis International Society; NSAIDs = Non-steroidal anti-inflammatory drugs; CRP = C-reactive protein.

**Table 2:** Characteristics of included studies focused on MMP levels in SpA patients.

Author	Type of MMP	Sample Type (Method)	Number of Cases Studied	Number of Controls Studied	Association with Diseases	Assessment of Disease Severity	Response to TNF Inhibitor Treatment	The TNF Inhibitor Studied
Liu et al. 2015 [52]	MMP3	Synovial tissue (IsH)	22 AS	22 Normal synovial tissue (Femoral head fracture)	Increased mRNA positive cells in cases	Correlation with CRP and BASDAI	NA	NA
Mou et al. 2015 [53]	MMP3	Serum (ELISA)	68 juvenile onset AS	32 HC	Increased in cases	Correlation with BASDAI and BASFI	NA	NA
Almodovar et al. 2014 [54]	MMP3	Serum (ELISA)	60 SpA	-	-	No correlation with CRP and BASDAI; correlation with the number of inflamed joints	NA	NA
Ramonda et al. 2013 [55]	MMP3	Serum (ELISA)	43 PsA	-	Increased in cases	Correlation with CRP	Baseline and reduction over time	E
Chandran et al. 2013 [66]	MMP3	Serum (ELISA)	40 PsA	-	-	NA	Baseline and reduction over time	E, A, G, I
Mattey et al. 2012 [56]	MMP1 MMP2 MMP3 MMP8 MMP9	Serum (ELISA)	180 AS	-	-	Correlation of all MMPs with CRP. Stronger association of MMP9 and MMP8 than MMP3 with BASDAI	NA	NA
Veidal et al. 2012 [69]	MMP2/MMP9	Serum (enzymatic activity)	40 AS	40 HC	Increased in cases	NA	NA	NA
Soliman et al.2012 [57]	MMP3	Serum (ELISA)	30 AS	30 HC	Increased in cases	Correlation with BASDAI, BASMI and BASFI	NA	NA
Pedersen et al. 2011 [58]	MMP3	Serum (ELISA)	60 SpA	333 HC	-	No correlation with radiographic progression	Reduction over time	E, A, I
Arends et al. 2011 [59]	MMP3	Serum (ELISA)	92 AS	-	-	Correlation with CRP and ASDAS in women, not in men	Moderate predictor of response in males with concomitant peripheral arthritis	E
Xia et al. 2011 [70]	MMP3	Serum (ELISA)	45 PsA	45 HC and 45 OA	Increased in cases	NA	NA	NA
Pedersen et al. 2010 [71]	MMP3	Serum (ELISA)	49 SpA	333 HC	Increased in cases	NA	Baseline	E, A, I
Chandran et al. 2010 [72]	MMP3	Serum (ELISA)	26 SpA	26 psoriasis, 26 HC	Increased in cases	NA	NA	NA
Van Kuijk et al. 2009 [67]	MMP3, MMP13	Synovial membrane (IHC)	24 PsA	-	-	NA	Reduction over time	A
Appel et al. 2008 [60]	MMP3	Serum (ELISA)	71 AS	-	-	Correlation with CRP, not with BASDAI	Reduction over time	A
Maksymowych et al. 2008 [61]	MMP3	Serum (ELISA)	82 AS	-	-	Correlation with CRP and with concomitant peripheral arthritis	Reduction over time	A
Wendling et al. 2008 [62]	MMP3	Serum (ELISA)	23 AS	21 HC	Increased in cases	No correlation with CRP and BASDAI	Reduction over time	E, A, I
Woo et al. 2007 [63]	MMP3	Serum (ELISA)	26 AS	-	-	Correlation with CRP	Reduction over time	E
Maksymowychet al. 2007 [64]	MMP3	Serum (ELISA)	217 AS	-	-	Correlation with CRP and with radiographic progression	NA	NA

Abbreviations: SpA = spondyloarthritis; AS = Ankylosing spondylitis; PsA = psoriatic arthritis; OA = osteoarthritis; HC = healthy controls; ELISA = enzyme linked immunosorbent assay; IsH = In situ hybridization; IHC = immunohistochemistry; CRP = C-reactive protein; BASDAI = Bath Ankylosing Spondylitis Disease Activity Index; BASFI = Bath Ankylosing Spondylitis Functional Index; BASMI = Bath Ankylosing Spondylitis Metrology Index; ASDAS = Ankylosing Spondylitis Disease Activity Score; A = adalimumab; E = etanercept; I = infliximab; G = golimumab; NA = not available.
